# Supplementary material for: Content-rich biological network constructed by mining PubMed abstracts
Source: BMC Bioinformatics. 2004 Oct 8;5:147. doi: 10.1186/1471-2105-5-147 (PMC528731; doi:10.1186/1471-2105-5-147)
Supplement: Additional File 5 — The original Chilibot query results of the term "long-term potentiation (LTP)" and 22 other terms, limiting the latest references analyzed to the years 1990, 1995, 2000, and 2004. [file 1471-2105-5-147-S5.bz2 › chilibotAdditionalFile5/ltp2000/html/PKC_AMPA.html]

 


 **PKC** and **AMPA** 
  
Found 56 abstracts in PubMed,  **30 abstracts were retrieved and analyzed**.  


---

 Search Google  |
 PDF files only 
|  EDU domain only 

---

**Interactive relationship** (e.g. stimulation, inhibition, etc)

- The results indicate not only that the  **AMPA**  receptor activation has a  **PKC**  requirement, but also that  **PKC**  amplifies maximal receptor activation by 100 microM kainate.  Ref: 9572298 J Neurochem, 1998
- In contrast to ACPD induced potentiation no evidence for an involvement of protein kinase C  **PKC**  is found in the CHPG induced potentiation of both NMDA and  **AMPA**  depolarization because the  **PKC**  blockers chelerythrine chloride or calphostin C did not antagonize this effect.  Ref: 10530818 Neuropharmacology, 1999
- All together, these results indicate that MAPK is implicated in the transmission of  **AMPA**  signaling to the nucleus and requires extracellular calcium, and PLC  **PKC**  activation.  Ref: 10095077 Brain Res Mol Brain Res, 1999
- We induced long term desensitization of  **AMPA**  receptors in rat cerebellar slices by 1 a 4 min bath application of quisqualate 0.1 mM or 2 a 15 min bath application of a protein kinase C  **PKC**  activator, phorbol 1 3 diacetate 0.5 microM or dibutyrate 0.6 microM, followed by a 4 min  **AMPA**  0.1 mM application.  Ref: 10456118 Learn MemLearn Mem, 1999
- Here we show that  **PKC**  phosphorylation of the  **AMPA**  receptor GluR2 subunit differentially modulates its interaction with the PDZ domain containing proteins GRIP1 and PICK1.  Ref: 11007883 J Neurosci, 2000
- Our results show an important role for  **PKC**  in regulating the function of both  **AMPA**  receptors and calcium channels in cultured retina cells.  Ref: 9572298 J Neurochem, 1998
- Here we report an interaction between  **AMPA**  receptor subunits and a single PDZ domain containing protein called PICK1 which is known to bind protein kinase C alpha  **PKC**  alpha.  Ref: 10340301 Neuropharmacology, 1999
- These results support biochemical observations indicating that  **PKC** , similar to calcium calmodulin dependent protein kinase II, has an important role in controlling synaptic strength via modulation of  **AMPA**  receptor function, potentially through the direct phosphorylation of the GluR1 subunit.  Ref: 9819284 J Neurophysiol, 1998
- In both the in vivo and the in vitro ischaemic models, administration of the  **AMPA**  receptor antagonist NBQX prevented the loss of  **PKC**  activity.  Ref: 9617487 Cell Signal, 1998
- Therefore, in contrast to cardiac tissue, ischemic preconditioning of neurons does not require activation of  **PKC**  and MAP kinase, and protection is maintained with substantial removal of extracellular calcium or blockade of the  **AMPA**  receptor.  Ref: 10320703 Brain Res, 1999

**Parallel relationship** (e.g. studied together, co-existance, homology, etc.)

- In addition, the protein kinase C  **PKC**  blockers staurosporine or chelerythrine chloride were able to antagonize the 1S, 3R ACPD induced potentiation of both NMDA and  **AMPA**  response, suggesting that the enhancing effect induced by Group I mGluRs is modulated by a  **PKC**  mediated mechanism.  Ref: 9294969 Neuropharmacology, 1997
- The  **AMPA**  receptor response to kainate was potentiated by phorbol 12 myristate 13 acetate, which specifically stimulates  **PKC** , and it was decreased by bisindolylmaleimide I, a selective inhibitor of  **PKC** , as well as by  **PKC**  down regulation.  Ref: 9572298 J Neurochem, 1998
- The functional modulation of the alpha amino 3 hydroxy 5 methyl 4 isoxazolepropionate  **AMPA**  receptors by protein kinase C  **PKC**  was investigated in cultures enriched in retinal amacrine like cells.  Ref: 9572298 J Neurochem, 1998
- In the analysis of the C terminal portion of the GluR2  **AMPA**  receptor, the phosphorylation sites of which had not hitherto been well clarified, we found the presence of at least three protein kinase C  **PKC**  phosphorylation sites.  Ref: 10775839 Brain Res Brain Res Protoc, 2000
- In the present study we have examined the role of  **PKC**  in tetanus induced LTP of  **AMPA**  receptor mediated synaptic transmission in hippocampal slices obtained from adult rats.  Ref: 11069602 Eur J Neurosci, 2000
- Using phosphorylation site specific antibodies, we found that induction of chem LTD produces a persistent dephosphorylation of the GluR1 subunit of  **AMPA**  receptors at serine 845, a cAMP dependent protein kinase PKA substrate... a substrate of protein kinase C  **PKC**  and calcium calmodulin dependent protein kinase II CaMKII.  Ref: 9856470 Neuron, 1998
- The phosphorylation of the glutamate receptor 1 GluR1 subunit of  **AMPA**  receptors by protein kinase A PKA, protein kinase C  **PKC** , and calcium calmodulin dependent protein kinase II CaMKII has been characterized extensively.  Ref: 10366608 J Neurosci, 1999
